# “Dragon man” prompts rethinking of Middle Pleistocene hominin systematics in Asia

**DOI:** 10.1016/j.xinn.2023.100527

**Published:** 2023-10-20

**Authors:** Christopher J. Bae, Wu Liu, Xiujie Wu, Yameng Zhang, Xijun Ni

**Affiliations:** 1 Department of Anthropology, University of Hawaii at Manoa, Honolulu, HI 96822, USA; 2 Institute of Vertebrate Paleontology and Paleoanthropology, Chinese Academy of Sciences, Beijing 10044, China; 3 Joint International Research Laboratory of Environmental and Social Archaeology, Shandong University, Qingdao 266237, China

Dear Editor,

Chibanian (Middle Pleistocene) hominin fossils that could not be easily assigned to *Homo erectus*, *H. neanderthalensis*, or *H. sapiens* have traditionally been assigned to an all-inclusive group: “archaic *H. sapiens*.” In an insightful observation of the Chibanian record almost four decades ago however, Tattersall railed against the use of the word “archaic” in this sense when referring to the human fossil record, as he justifiably noted that no other biological organism has the word “archaic” attached to it.^1^ For example, no one refers to an earlier version of *Canis domesticus* as “archaic” *C. domesticus*. The ancestor of the domestic dog is, and always has been, considered to be *C. lupus*. In Tattersall’s opinion, it would seem that these “archaic *H. sapiens*” fossils should be assigned to one or more formal taxonomic names. As such, terms such as “archaic *H. sapiens*,” “mid-Pleistocene *Homo*,” and “Middle Pleistocene *Homo*” have always been considered to be wastebasket taxa that include way too much morphological variability for one proposed taxonomic group. Continuing to use wastebasket taxa only hinders any attempts to understand true phylogenetic and evolutionary relationships.

In more recent decades, there has been a push, primarily by Western paleoanthropologists, to assign these archaic *H. sapiens* fossils to another all-inclusive taxon: *H. heidelbergensis*.^2^^,^^3^ This would include all of the fossils from Europe, Africa, and East Asia. However, some researchers have questioned the utility of *H. heidelbergensis* representing all Chibanian hominins not named *erectus*, *neanderthalensis*, or *sapiens*.^4^ At least part of the reason is due to the recent publication of new Chibanian taxa in places such as South Africa (*H. naledi*)^5^ and China (*H. longi*).^6^ Furthermore, questions have long been raised about trying to assign the Chinese fossils to the *H. heidelbergensis* hypodigm, given that the hominins from eastern Asia look quite different from similarly aged fossils from western Eurasia and Africa (Figure 1 inset).

**Figure 1:**
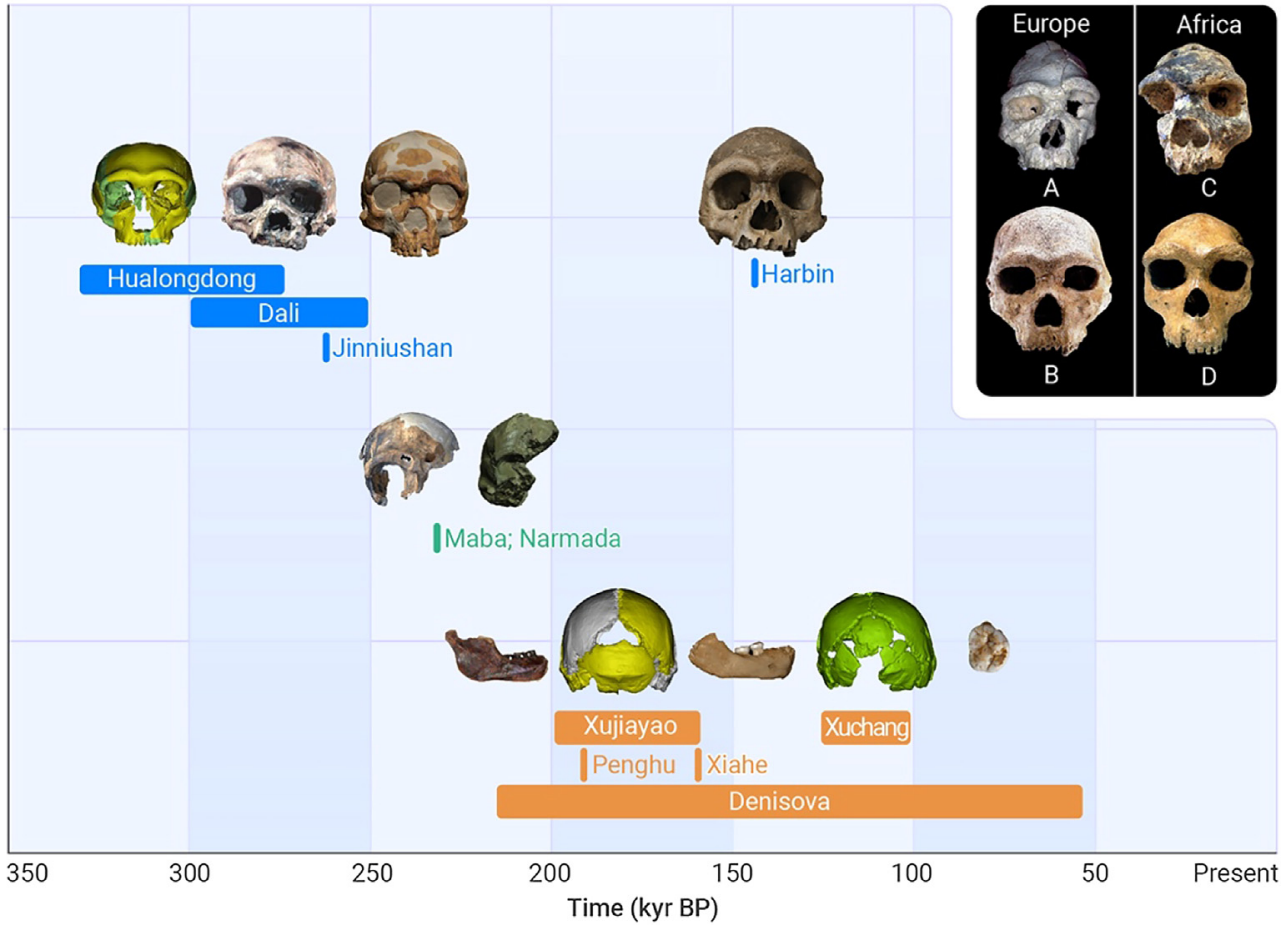
Proposed groupings for the Chinese/Asian fossils Group 1 includes Harbin, Dali, Jinniushan, and Hualongdong; group 2 includes Maba and Narmada (Hathnora); and group 3 includes Xujiayao, Xuchang, Xiahe, Penghu, and Denisova. Inset: for comparative purposes, these are representative Chibanian fossils from Europe (A, Arago; B, Petralona) and Africa (C, Bodo; D, Kabwe).

## Available nomina

The Chinese Chibanian hominin fossil record is taking on an increasingly important role in this debate. An important observation from this time period is that there is a greater amount of hominin morphological variability present than originally hypothesized. How to organize all of this variation is the next logical step in this process. Unfortunately, because Chinese (and many westerners familiar with the Chinese record) paleoanthropologists have traditionally viewed the Chibanian hominin fossil record as transitional between *H. erectus* and *H. sapiens* these fossils have long been referred to simply as “archaic” or “early” *H. sapiens*. This may be part of the reason that almost a century passed between Davidson Black’s formal announcement of *Sinanthropus pekinensis* in 1928 and *H. longi* in 2021. But is it true that no Chinese Chibanian hominin taxonomic names have been formally published and available between *S. pekinensis* and *H. longi*? We delve more deeply into this question, particularly given how important the Chinese record is to these debates in paleoanthropology. In fact, with the recent announcement and description of the new *H. longi* type specimen (Harbin) by Ni et al.,^6^ other possible taxonomic names seem to have been raised as possibly available, and importantly, would have priority (e.g., *H. daliensis*, *H. mapaensis*).

A good example of this is that an argument has been made (on social media) that on the basis of morphological similarities, the type specimen of *H. longi*, the Harbin cranium, should be assigned to *H. daliensis*. The justification for this argument is that the Dali specimen was supposedly published as a new species and would have priority. We conducted an extensive review of this argument and conclude that Xinzhi Wu had proposed the Dali cranium as a possible subspecies of *H. sapiens*. In the original 1981 publication in English, Wu wrote that “it is suggested that Dali cranium probably represents a new subspecies, *Homo sapiens daliensis*” (p. 538).^7^ Even in the original Chinese version of the paper however, Wu used the term “probably” to describe Dali as a possible new subspecies.^7^ Furthermore, in all of Wu’s follow up publications, *H. daliensis* does not appear as a separate species. In other words, Wu did not discuss or consider Dali in formal taxonomic terms and certainly not as a new subspecies or species in his more recent publications. In fact, other than in his 1981 paper, Wu always referred to the Dali fossil as “archaic” *H. sapiens*. It is our experience, which includes many direct discussions with him, that Wu strongly believed that all Chinese Chibanian hominins are transitional forms between *H. erectus* and modern *H. sapiens* and that Dali is the closest to modern *H. sapiens*. This raises the question, then, of who started to use “*H. daliensis*” as a species name. To the best of our knowledge, Dennis Etler^8^ may be the first researcher to mention *H. daliensis*. However, even in Etler’s paper, the name is used only once in a figure, and is never mentioned in the main text. It is unclear whether he intended to raise Dali to the species level. Although we do not know exactly what Etler was thinking when he included *H. daliensis* in his figure, it would seem that he may have simply been replacing “archaic *H. sapiens*” with a taxonomic name, more like a placeholder of sorts, something that is frequently seen in European and African Chibanian research. It should be noted that the International Commission on Zoological Nomenclature (ICZN) has clear instructions on nomina that have been proposed conditionally: “A new name or nomenclatural act proposed conditionally and published after 1960 *is not thereby made available*” (ICZN 1999, p. 19; emphasis added). *H. daliensis* was never formally presented as a new species, and given current ICZN guidelines, it would seem the name is currently unavailable.

Another possible hominin nomen that has appeared is *H. erectus mapaensis*,^9^ based on of the important Maba cranial fossil. From a review of Woo and Peng’s^10^ original Maba paper, however, it would appear that the original analysts did not attempt to assign a species or even a subspecies name to the Maba specimen. Indeed, the last two sentences of the original paper read as follows: “The Maba hominin is an early archaic *Homo sapiens* fossil that fills a gap in our understanding of human evolution from *H. erectus* to archaic *H. sapiens*. This specimen is therefore a key piece of evidence for understanding this important transition in human evolution” (our translation from the original Chinese text). Thus, it would appear that Kurth^9^ may be the first person to mention *H. erectus mapaensis*, as he wrote, “perhaps *H. e*. *mapaensis* should be added as a later (end middle Pleistocene?) layer from the south” (our translation from the original German text). However, given the tentative proposed subspecies, it would suggest that *H. erectus mapaensis* should also be considered unavailable following ICZN guidelines.

Given the unavailability of *H. daliensis* and *H. mapaensis*, this raises the question as to what name(s) we should assign to the Chinese, and broader Asian, Chibanian hominin fossil record.

## Taxonomic groupings

How many Chibanian hominin taxonomic groups are present in China? At a broad level, the phylogenetic analyses of Ni et al.^6^ showed that most of the Asian Middle Pleistocene hominins belong to a monophyletic group, suggesting that these hominins may be assigned to a single taxonomic group. However, given the growing range of morphological variation in the Asian Chibanian hominin fossil record, it is important to start to organize the fossils into different morphotypes as it is clear several different types are present. The proposed groupings depicted in Figure 1 are based on our knowledge of the record today.

Broadly speaking, at this moment, it would be better to keep Dali and Harbin separate, as there are no detailed comparative studies to justify lumping them. However, following further analyses, it may be possible Dali and Harbin could be assigned to the same taxon. If this occurs, then Dali should be assigned to the *H. longi* hypodigm. On the basis of comparative morphometrics, Jinniushan and Hualongdong may eventually be included in the *H. longi* hypodigm as well. Xujiayao and Xuchang, and on the basis of dentognathic comparisons Xiahe, Penghu, and the Denisovans, should be assigned to something else forthcoming, though Ni et al.^6^ may argue that Xiahe belongs in *H. longi*. The Maba and Narmada (Hathnora) crania have long been considered to be similar enough to be assigned to their own group as well. More detailed evaluation of these groupings is certainly warranted, though such analyses fall outside the scope of the current study. Regardless, it is clear that there is a growing range of variation in the Asian hominin fossil record. What role this record plays in broader debates is only beginning to be more fully understood.

## Moving forward

The growing Chibanian hominin fossil record across Eurasia and Africa is raising a number of interesting questions related to its variability and how these different types are related to each other. In this regard, the Chinese fossil record plays a critical role in developing a better understanding of this debate. As part of these contributions from China, it is important to evaluate which hominin nomina are valid or not. It would appear that besides *H. longi*, there are probably at least two other types (Xujiayao/Xuchang and Maba) represented by the Chinese hominin fossil record. *H. sapiens daliensis*, *H. daliensis*, *H. erectus mapaensis*, and *H. mapaensis* are not available and should be removed from consideration.
